# Seed Pubescence and Shape Modulate Adaptive Responses to Fire Cues

**DOI:** 10.1371/journal.pone.0159655

**Published:** 2016-07-20

**Authors:** Susana Gómez-González, Fernando Ojeda, Patricio Torres-Morales, Jazmín E. Palma

**Affiliations:** 1 Centre for Science and Resilience Research (CR²), Universidad de Chile, Santiago de Chile, Chile; 2 Departamento de Biología-IVAGRO, Universidad de Cádiz, Campus Río San Pedro, Puerto Real, Spain; 3 Instituto de Ciencias Ambientales y Evolutivas, Universidad Austral de Chile, Valdivia, Chile; Henan Agricultural University, CHINA

## Abstract

Post-fire recruitment by seeds is regarded as an adaptive response in fire-prone ecosystems. Nevertheless, little is known about which heritable seed traits are functional to the main signals of fire (heat and smoke), thus having the potential to evolve. Here, we explored whether three seed traits (pubescence, dormancy and shape) and fire regime modulate seed response to fire cues(heat and smoke). As a model study system, we used *Helenium aromaticum* (Asteraceae), a native annual forb from the Chilean matorral, where fires are anthropogenic. We related seed trait values with fitness responses (germination and survival) after exposure to heat-shock and smoke experimental treatments on seeds from 10 *H*. *aromaticum* wild populations. We performed a phenotypic selection experiment to examine the relationship of seed traits with post-treatment fitness within a population (adaptive hypothesis). We then explored whether fire frequency in natural habitats was associated with trait expression across populations, and with germination and survival responses to experimental fire-cues. We found that populations subjected to higher fire frequency had, in average, more rounded and pubescent seeds than populations from rarely burned areas. Populations with more rounded and pubescent seeds were more resistant to 80°C heat-shock and smoke treatments.There was correlated selection on seed traits: pubescent-rounded or glabrouscent-elongated seeds had the highest probability of germinating after heat-shock treatments. Seed pubescence and shape in *H*. *aromaticum* are heritable traits that modulate adaptive responses to fire. Our results provide new insights into the process of plant adaptation to fire and highlight the relevance of human-made fires as a strong evolutionary agent in the Anthropocene.

## Introduction

Fire has influenced terrestrial plant communities since their appearance on earth [1–3] and has played a key role in the geographic expansion of large groups of plants, such as angiosperms during the Cretaceous [4] and C_4_ grasses during the Miocene [5]. However, despite its unequivocal influence on global vegetation at geological time scale, establishing whether plant traits with functional relevance in fire-prone environments have been selected by fire remains an open question (e.g., [6, 7]).

Studies performed on Mediterranean-type climate (MTC) regions have provided a great body of evidence on the role of fire in shaping the evolution of plant traits. Since the MTC emerged in the five Mediterranean regions of the world (at least since the Pliocene), processes of convergent evolution have shaped similar plant formations [8]. All MTC regions, except Central Chile, have been subjected to natural fire regimes ever since [8, 9] and therefore fire has played a role in the evolutionary history of their floras [10]. Plant species in these ecosystems show fire-response traits such as resprouting [11], fire-stimulated seed release (i.e. serotiny [12, 13]), thick bark [14], fire-stimulated flowering [15], flammability [16, 17] and fire-cued seed germination through heat-shock and/or smoke [18–21]). Although these traits are considered adaptations to fire ([22], and references therein), some authors have argued that their evolution might have been caused by other selective forces (e.g. drought, herbivory) and they should hence be regarded as exaptations [6].

In this sense, both macro- and microevolutionary approaches can been used to assess the importance of fire on the evolution of plant traits [22]. Character mapping on phylogenies, for example, have shown that the appearance of thick bark, serotiny and epicormic resprouting in the genera *Pinus*, *Banksia* and *Eucalyptus* are linked to different changes in the fire regime between 125 and 70 My bp [14, 23, 24]. The role of fire on the evolution of seed persistence (fire-stimulated seedbanks) has been also demonstrated by using phylogenetic methods [25–27].

In contrast to the large amount of macroevolutionary evidence, the role of fire in shaping plant traits remains little explored from a microevolutionary (within-species) perspective [28–29]. This perspective consists of proving that evolution by natural selection of traits occurs in wild populations. It is achieved by showing the existence of trait variability within populations, the functionality of the trait against the selective agent and its heritability, and also, by searching for divergence in adaptive traits among populations along ecological gradients [30, 31]. For example, flammability in *Ulex parviflorus* is a functional trait in fire-prone environments, it is heritable and it varies among populations subjected to different fire frequencies, thus indicating that fire is linked to its evolution [16, 17].

Regarding seed traits, the knowledge on how fire changes the frequency of functional and genetically-based seed traits in wild populations is still poor, with contrasting results. For instance, Shryock *et al*. [32] found that species with smaller seeds were more frequent after fire in the Mojave Desert. By contrast, seed shape (not seed size) was related with fire in species from temperate grasslands [33]. To our knowledge, only the study by Gómez-González *et al*. [28] has demonstrated the existence of fire-driven phenotypic selection in heritable seed traits. They showed that populations inhabiting areas under frequent, recurrent fires had more pubescent and rounded seeds than those from populations under low fire frequency. In a phenotypic selection experiment within a single population (burning litter layer over seeds), germination was higher in more elongated and pubescent seeds [28] (demonstrating the adaptive value of the traits). Finally, they showed that both seed traits were heritable, highlighting their potential to evolve under increasing fire frequency. However, the mechanisms underlying the functionality of seed pubescence and shape under recurrent fire are still unknown. Gómez-González *et al*. [28] proposed that pubescence might provide some thermal insulation and rounded seed might be favoured because it might be easier for them to be buried in the field, not because they were more resistant to fire. In this sense, phenotypic selection experiments evaluating the contribution of these traits to fitness response after heat-shock and smoke are needed, in order to demonstrate that they are adaptations against the specific signals of fire.

Seed dormancy is a heritable trait that can evolve by natural selection [34]. Seeder (i.e. post-fire recruiting) plants from fire-prone ecosystems form persistent seed-banks and germination is triggered by fire-related cues (e.g. heat, smoke; [21]). Generally, the higher the dependence of fire cues for recruitment the larger the proportion of dormant seeds [20]. Moreira & Pausas [35] proposed that fire, rather than summer heat, shapes the temperature thresholds breaking physical seed dormancy in Mediterranean species. This conclusion was achieved after comparing the germination under heat-shock vs. long-term exposure to summer-simulated temperatures in several populations of six obligate seeder species. However, the responses to fire cues have not been associated with the intraspecific variation of seed dormancy in order to test an adaptive hypothesis, and thus, the role of fire as selective agent shaping this trait remains unclear.

Here, we used *Helenium aromaticum* (Asteraceae), a native annual forb from the Chilean Mediterranean matorral, as a model study system to ascertain whether fire drives the rapid evolution of seed traits through the effects of heat and/or smoke. Compared to other MTC regions, recurrent fire in Central Chile is a recent phenomenon related to the history of human colonization [10, 36–38]. Although natural fires seem to have been common during the Miocene [39], the reduction of fire after the rising of the Andes probably produced a relaxation or loss of fire-related traits in the Chilean flora [10]. Therefore, this is the only MTC region considered not to have been shaped by fire [8, 9, 40, 41]. The fact that fire is a recent, anthropogenic disturbance on *H*. *aromaticum* makes this species an adequate model to explore the role of fire on the evolution of plant traits at an ecological time scale [28]. This question was initially addressed with the same study species by Gómez-González *et al*. [28], but here we delve into the mechanisms underlying adaptive fire responses by analyzing their association with fire-specific cues, viz. heat-shock and smoke.

We hypothesized that seed pubescence, shape, and dormancy are shaped by fires cues in *H*. *aromaticum*. If so, we expect that seed germination and survival (fitness measures) after either heat-shock or smoke cues would be associated with the expression of these traits across populations subjected to different fire frequencies and across individuals within a single population (phenotypic selection by fire cues). Besides, if fire breaks seed dormancy, then germination after fire cues should increase with the proportion of dormant seeds. We also hypothesized a positive relationship between fire frequency and the seed responses to fire-cues. Since recurrent fires are novel in this area, the increase of fire frequency should select for higher seed resistance to heat and/or smoke. Finally, we expect that the correlation between seed traits (pubescence and shape) and fire frequency showed in Gómez-González *et al*. [28] (seeds collected in 2009) will be maintained four years later (seeds from 2013), thereby confirming the hypothesis of heritable fire-selected traits.

## Materials and Methods

### Study species

*Helenium aromaticum* (Asteraceae) is an annual forb native to Chile and distributed along the Chilean MTC region (Central Chile) [42]. It generally inhabits open patches in dry hills, flowering in December and producing fruits from January to March. Dispersal units are achenes (i.e. indehiscent, one-seeded dry fruits) but, for simplicity, we will refer to them as seeds. They are coniform and pubescent, with a short, rudimentary pappus [42], not functional for wind dispersal. Main dispersal mechanism is simple gravity (barochory), but there is probably a secondary dispersal by rainwater.

### Source populations, fire regime and seed sampling

**Fig 1:**
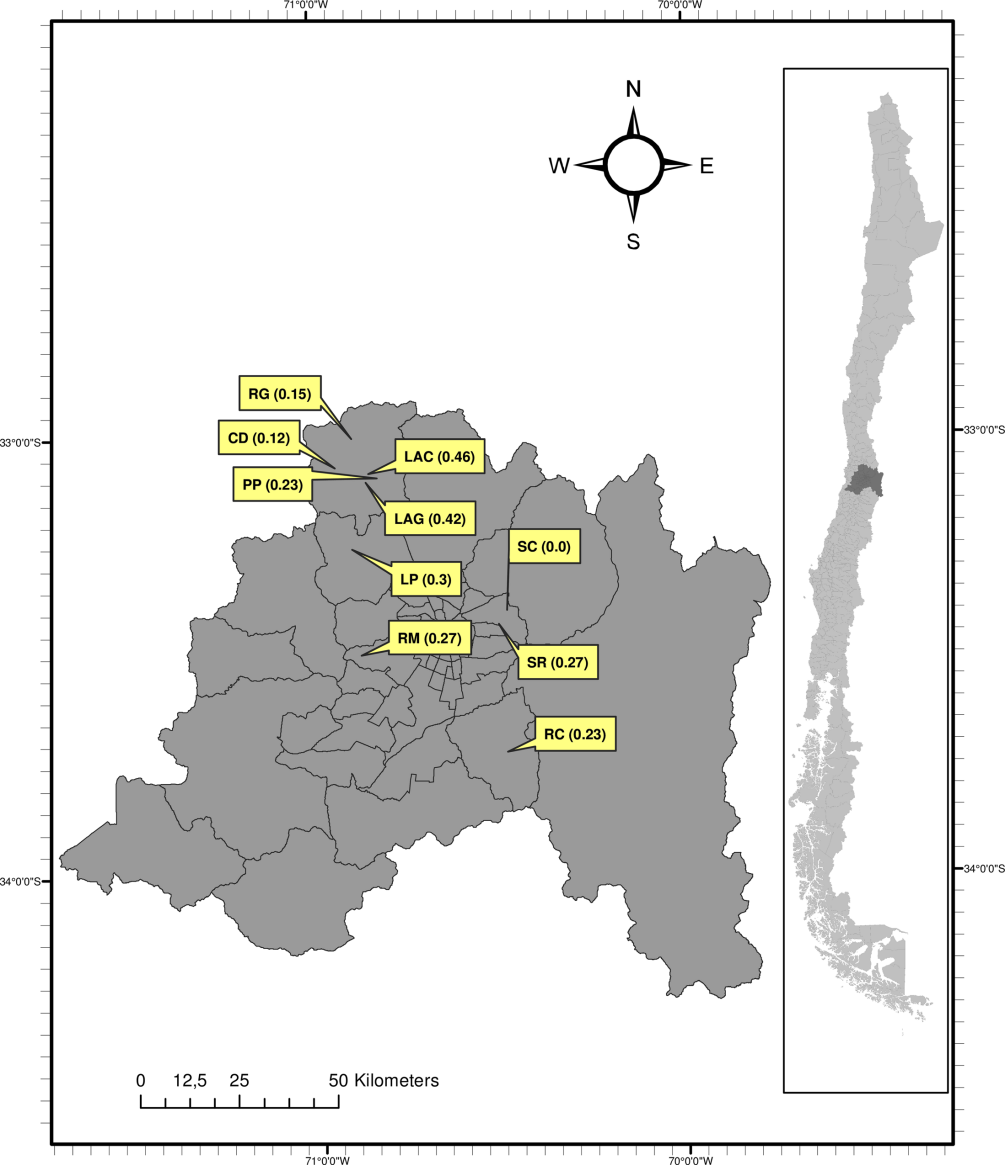
Location of the 10 *H*. *aromaticum* populations in matorral plant communities along the Metropolitan County (Central Chile). Fire frequency of each population is indicated in parentheses (no. fires·y^−1^, see site description in S1 Table and Gómez-González *et al*. [28] for methodological details). CD: Cuesta La Dormida; LAC: Los Aromos (crop area); LAG: Los Aromos (grazing area); LP: Lampa; PP: Punta de Peuco; RC: Río Clarillo; RG: Rungue; RM: Rinconada Maipú; SC: San Carlos; SR: San Ramón.

This study was performed on seeds from 10 natural populations of *H*. *aromaticum* located in matorral communities within the Metropolitan County (Central Chile) (Fig 1), for which fire frequency was previously determined (see Gómez-González *et al*. [28] for methodological details). Fire return interval in the study sites ranged from no fires in at least 26 years to one fire every 2–3 years (S1 Table). Climate in the Metropolitan County is Mediterranean-type, characterized by rainy winters and long dry summers. Annual rainfall ranges from 300 to 500 mm. Mean annual temperature is 18°C, with maximum temperatures above 30°C in summer and minimum temperatures below 0°C in winter [43]. Vegetation is patchy and dominated by sclerophyllous woody species such as *Cryptocarya alba*, *Lithraea caustica*, *Quillaja saponaria*, *Peumus boldus*, and some xerophytes such as *Baccharis* spp. and *Acacia caven*.

In January 2013, we collected mature seeds from *H*. *aromaticum* plants growing in open, sunny microhabitats (to avoid different microclimatic conditions among populations) at each study site. We selected plant individuals that were at least 10m apart from each other to ensure genetic variability. We collected around 30 individuals per population (except for LAG population, where only eight individuals were found, and LP population where 68 were collected for the phenotypic selection experiment as described below). All seeds of each individual plant were placed in a labelled paper bag. The National Forest Corporation (CONAF, Chile) and the Association Parque Cordillera gave us the permission to collect seeds from Río Clarillo National Reserve (Pirque, Chile), San Carlos de Apoquindo Park (Las Condes, Chile), and Aguas de Ramón Natural Park (La Reina, Chile) (Fig 1, S1 Table).

### Fire-cue experiments and seed traits measure across populations

We randomly selected 10 individuals (i.e. 10 seed bags) from each population (except for LAG population, where only eight individuals where found). From each individual, 150 seeds were placed in five sterile Petri dishes with absorbent paper (30 seeds per Petri dish) and subjected to five treatments: exposure to (1) 80°C during 5 min, (2) 100°C during 5 min, (3) 120°C during 5 min, (4) cold aerosol smoke during 30 min, and (5) no-treatment (control). Then, we had 10 samples (individuals) of 30 seeds each, per treatment and population. These treatments were based on the soil temperature reached after experimental fire in the matorral [44] and the ranges of values used in studies from other MTC regions (e.g. [21]). Heat shock treatments took place in a convection oven, whereas the smoke exposure treatment was applied in a sealed plastic chamber connected to a bee smoker by a long plastic tube. Smoke was produced by burning a mixture of dry litter of *L*. *caustica* and *C*.*alba*, the most abundant tree species at the sites where *H*. *aromaticum* plants were sampled. The temperature inside the chamber was monitored throughout the experiment and it was always less than 25°C. Treatments were applied in groups of 10 Petri dishes (one individual of each population). Then, Petri dishes were kept moist by watering to saturation with distilled water and placed in a germination chamber under favourable conditions for germination (simulating autumn temperatures: 12h light at 20°C and 12h dark at 10°C). The 500 Petri dishes (10 individuals x 10 populations x 5 treatments) were watered and checked daily for seedling emergence for 36 days. After that period, we determined the viability of non-germinated seeds using the tetrazolium test (TTC 1% in phosphate buffer, pH 7.3, and 24h in darkness).

We calculated the percentage of seed germination of each individual as the number of germinated seeds divided by the total number of seeds in the Petri dish. We calculated the percentage of seed survival of each individual as the number of germinated seeds plus the number of non-germinated viable seeds divided by the total number of seeds in the Petri dish. From the same plant individuals in which heat and smoke assays were conducted (10 individuals x 10 populations; see above), we randomly selected other seeds to measure seed pubescence, shape and dormancy. For this, seeds were photographed under a binocular stereoscope (5x) and images were analyzed with SigmaScan® Pro (Systat Software, Inc.). Seed shape was estimated in five seeds as the length-to-width ratio of the seed surface (ignoring the pappus). Hence, more elongated seeds had higher values of this ratio. Seed pubescence was visually estimated in five seeds by assigning a category of pubescence from one to six (see in Gómez-González *et al*. [28] the criteria for these categories). When seed pubescence was intermediate between two categories, we added 0.5 to the lowest value. As we calculated the mean pubescence per individual (n = 5 seeds), the response variable was continuous (as in Gómez-González *et al*. [28]). Dormancy was estimated as the percentage of non-germinated viable seeds in control samples (of 30 seeds each) of the experiment described above.

### Phenotypic selection experiment (adaptive response to fire-cues)

To perform the phenotypic selection experiment we selected a population with an intermediate level of fire occurrence; Lampa (LP; Fig 1), as in the previous study of Gómez-González *et al*. [28]. We collected mature seeds from 68 plant individuals in January 2013. Some seeds of each plant individual were used for seed trait measures as described above (pubescence, shape and dormancy), and 100 seeds from the same plant individual were used in the selection experiment. Fifty seeds of each plant individual were placed on Petri dishes and subjected to two treatments of heat (80°C and 100°C during 5 min), and after that, to the germination essay as described above (68 individuals x 50 seeds x 2 treatments). We used only heat-shock as fire cue because there were not enough seeds for additional treatments. The probability of germination and survival of each individual after each heat treatment was used as a measure of fitness.

### Statistical analyses

We used Generalized Linear Mixed Models (GLMMs) to assess the effect of fire-cue treatments on seed germination and survival of *H*. *aromaticum* across populations (10 individuals x 10 populations), and how these effects vary with seed trait expression. This is an analysis of covariance with binomial distribution data (percentage of germination and survival), where fire-cue exposure is the categorical independent variable (four levels: Control, 80°C, 100°C and smoke), seed traits (pubescence and shape) are the independent continuous variables, and the population is the random factor (blocks). Likelihood ratio tests (LRT) were used to assess the interaction between factors (traits x fire cues) and then the effect of factor levels was evaluated with z-tests. We also used GLMM analysis to assess the relationship of dormancy (independent continuous variable) with the percentage of germination after each fire-treatment (80°C, 100°C and smoke; z-tests).

We performed Generalized Linear Models (GLZs) and LRT to assess whether the effect of fire-related cues on the mean percentage of germination and survival (n = 10 populations) varied with the fire frequency of *H*. *aromaticum* habitats. We used correlation analyses (Pearson correlation for pubescence and shape, and Spearman-Ranks correlation for dormancy) to assess the relationship between seed traits and fire frequency across populations in 2013.

To test the adaptive hypothesis (phenotypic selection experiment) within the Lampa population, the multiple regression approach was used [45]. Selection gradients were calculated by regressing the probability of seed germination or survival on standardized traits (mean = 0, SD = 1). We used generalized linear models and t-tests to determine the significance of the estimated parameters; linear selection gradients (ß_i_) assess the magnitude of directional selection acting on the trait’s mean, quadratic gradients (γ_i_) assess the form (stabilizing or disruptive) of the selection function, and correlational gradients (γ_ij_) reveal particular combinations of trait states that are selected together [45]. Before performing the selection analyses, we ruled out the co-linearity between traits using Pearson correlations (S2 Table).

All statistical analyses were performed with R software [46] and lme4 package for GLMMs [47].

## Results

We found no germination and negligible seed survival after the 120°C heat-shock treatment, so these data were eliminated from the analyses, and we concluded that *H*.*aromaticum* seeds are not resistant to such a high temperature.

**Fig 2:**
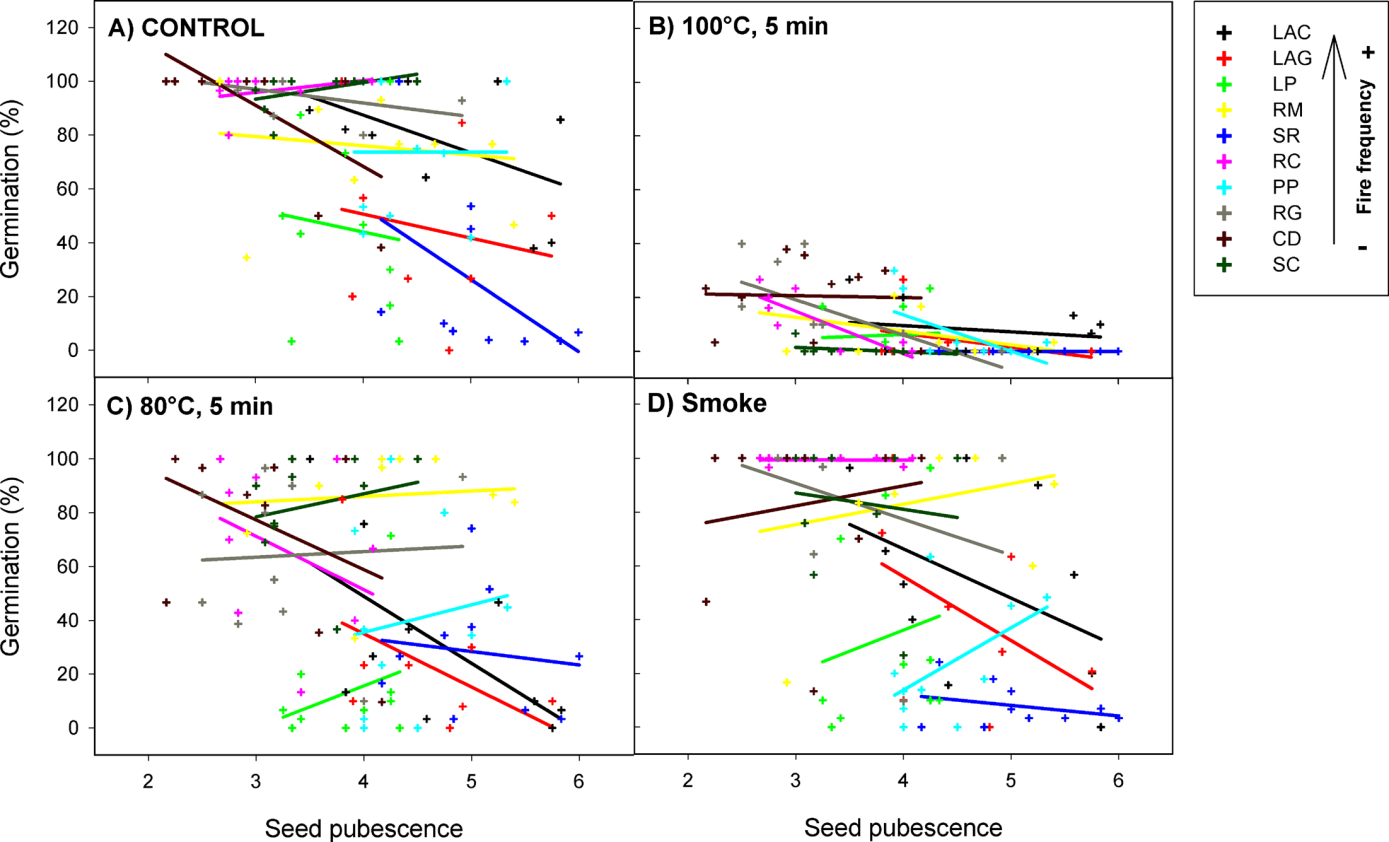
Interactive effects of fire cues and seed pubescence on the percentage of germination of *H*. *aromaticum* populations. Crosses represent plant individuals and there is one regression line per population (with different colours). Population codes in the box are in decreasing order of fire frequency (see codes in Fig 1 legend).

**Fig 3:**
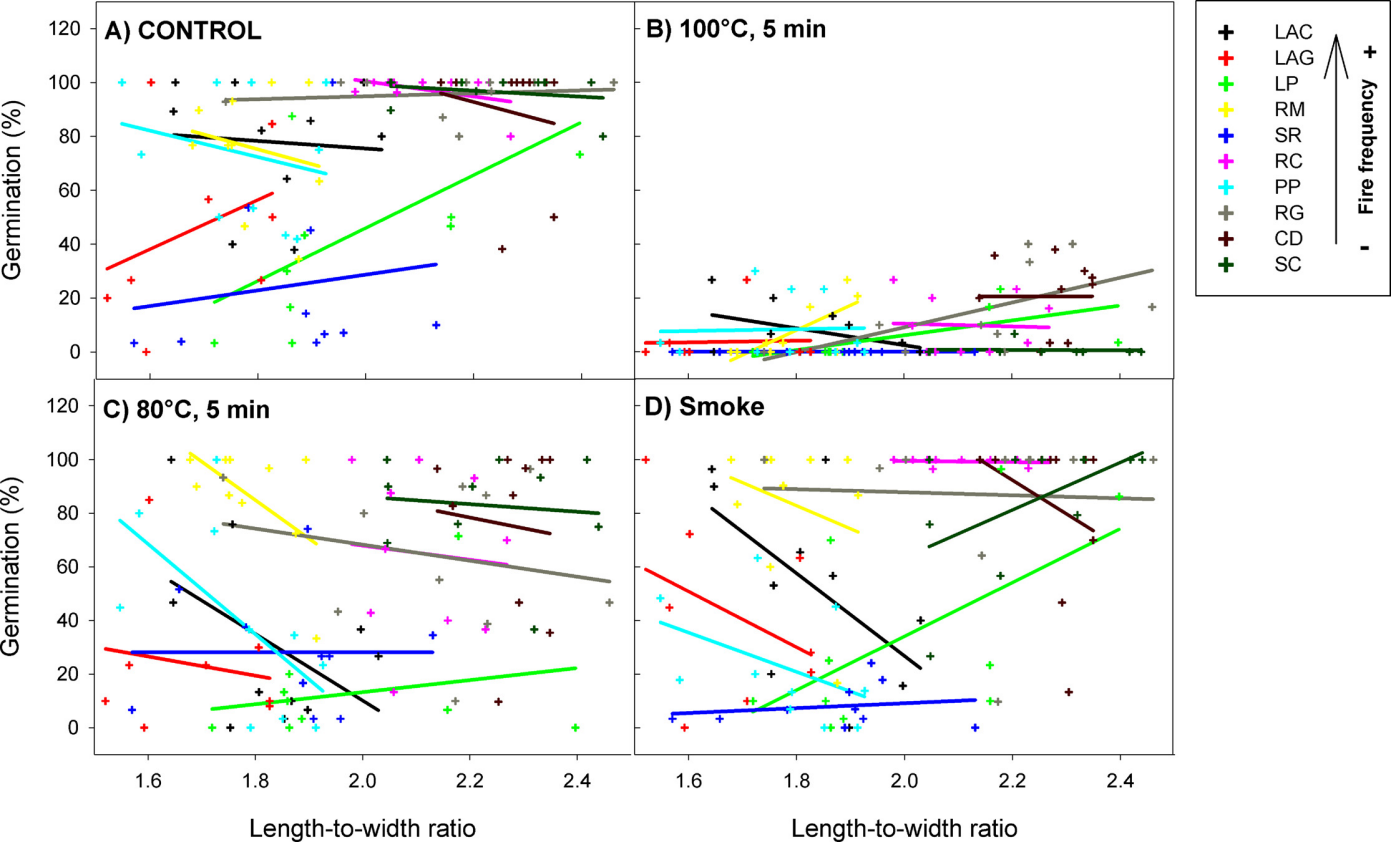
Interactive effects of fire cues and seed shape (length-to-width ratio) on the percentage of germination of *H*. *aromaticum* populations. Crosses represent plant individuals and there is one regression line per population (with different colours). Population codes in the box are in decreasing order of fire frequency (see codes in Fig 1 legend).

All fire-related treatments (smoke80°C and 100°C) significantly reduced the percentage of seed germination and survival from all *H*. *aromaticum* populations (Table 1). However, this effect was different depending on the expression of seed pubescence (germination χ^2^ = 38.30, P < 0.001; survival χ^2^ = 98.83, P < 0.001; Fig 2) and shape (germination χ^2^ = 79.59, P < 0.001; survival χ^2^ = 75.72, P < 0.001; Fig 3). Specifically, the effect of the 80°C heat-shock treatment on seed germination and survival became less negative (with respect to the control) with increasing seed pubescence (significant positive slope for *80°C x pubescence* interaction; Table 1; Fig 2A and 2C). In the same way, the effect of smoke on seed survival became less negative on pubescent seeds (significant positive slope for *smoke x pubescence* interaction; Table 1; S1 Fig). The effect of 80°C and 100°C heat shock on seed germination was more negative as seeds were more elongated (significant negative slopes for *80°C x shape* and *100°C x shape* interactions; Table 1; Fig 3A, 3B and 3C). Similarly, the effect of 80°C and smoke treatments on survival was more negative in elongated than in rounded seeds (significant negative slopes for *80°C x shape* and *smoke x shape* interactions; Table 1; S2 Fig). Regarding all treatments together, pubescence reduced the percentage of seed germination and survival of all *H*. *aromaticum* populations, while seed shape had no effects on these response variables (Table 1).

**Table 1:** GLMM analyses showing the interaction between fire cues and seed traits on seed germination and survival of *H*. *aromaticum*.

Source of variation	Estimate (*β*)	Std. Error	*z*-value	*P*-value
**Seed germination (%)**				
Smoke	-0.75	0.06	-11.52	**<0.001**
80°C	-1.22	0.07	-18.48	**<0.001**
100°C	-4.16	0.09	-46.05	**<0.001**
Pubescence	-0.28	0.03	-8.74	**<0.001**
Pubescence x Smoke	0.11	0.08	1.36	0.173
Pubescence x 80°C	0.44	0.07	5.67	**<0.001**
Pubescence x 100°C	0.16	0.11	1.50	0.132
Shape (length:width)	-0.20	0.15	-1.40	0.161
Shape x Smoke	-0.29	0.30	-0.98	0.325
Shape x 80°C	-2.19	0.29	-7.64	**<0.001**
Shape x 100°C	-1.06	0.39	-2.68	**0.007**
**Seed survival (%)**				
Smoke	-0.69	0.14	-4.97	**<0.001**
80°C	-1.29	0.13	-9.95	**<0.001**
100°C	5.66	0.13	-44.00	**<0.001**
Pubescence	-0.11	0.035	-3.23	**0.001**
Pubescence x Smoke	0.63	0.15	4.06	**<0.001**
Pubescence x 80°C	0.66	0.14	4.60	**<0.001**
Pubescence x 100°C	-0.25	0.15	-1.73	0.083
Shape (length:width)	-0.02	0.09	-0.29	0.774
Shape x Smoke	-3.31	0.61	-5.43	**<0.001**
Shape x 80°C	-2.34	0.56	-4.17	**<0.001**
Shape x 100°C	-0.11	0.55	-0.20	0.844

Significant *P*-values are are highlighted in bold (*P* < 0.05; GLMM and *z*-test).

Seed dormancy in all *H*. *aromaticum* populations was negatively related to the percentage of seed germination after fire-related cues (smoke: *z* = -8.79, *P* < 0.001; 80°C: *z* = -13.68, *P* < 0.001; 100°C: *z* = -3.57, *P* < 0.001; S3 Fig). That is, our treatments did not break seed dormancy in the study species.

**Fig 4:**
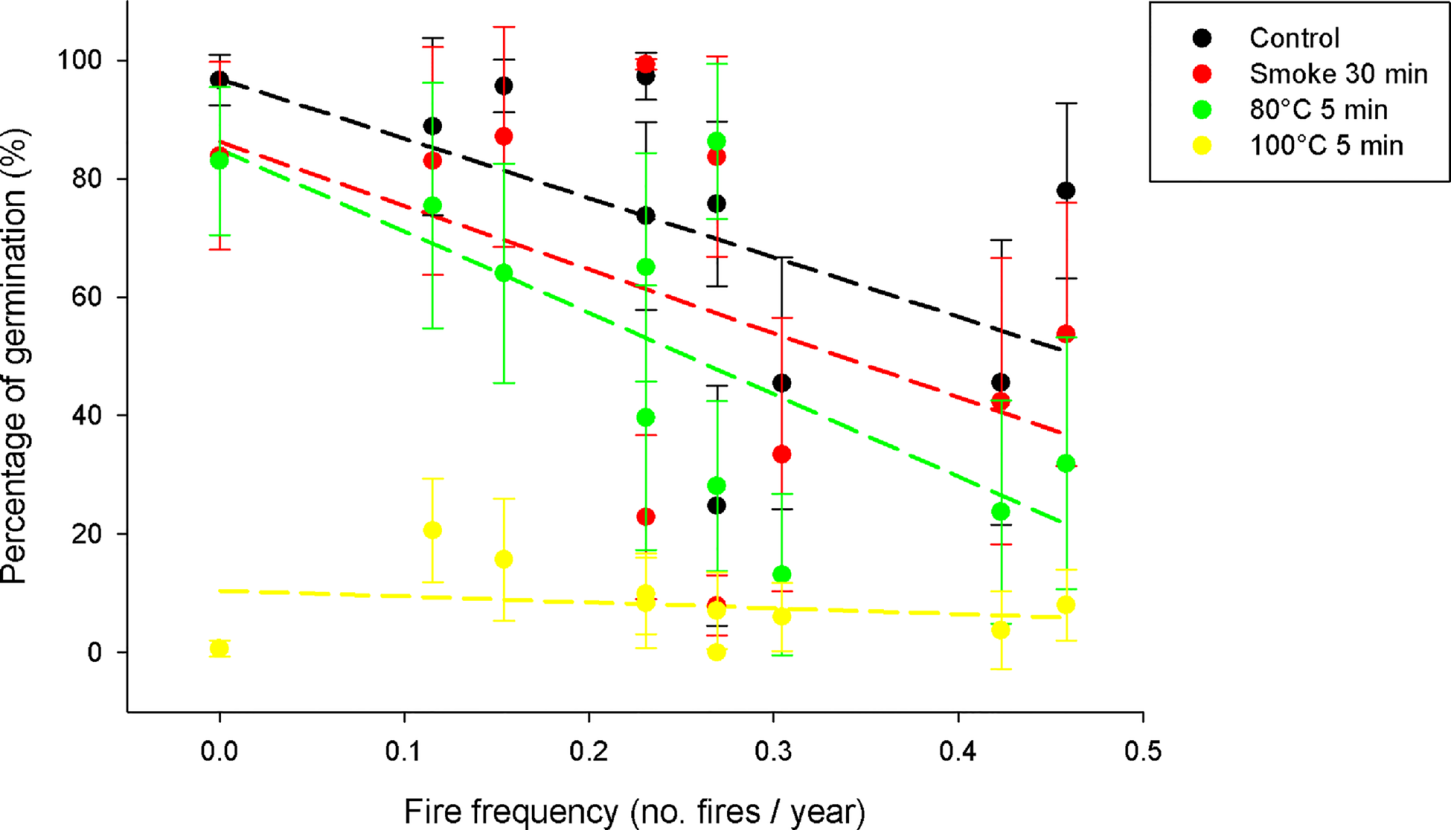
Effect of fire cues and fire frequency on seed germination of *H*. *aromaticum* populations. Dots represent the mean value of populations and error lines are 2SE (n = 10 individuals). Lines of different colours are the regression lines of the treatments.

Fire frequency was negatively associated with the percentage of seed germination (*t* = -3.56, *P* = 0.001; Fig 4) and was not related to seed survival (*t* = 0.56, *P* = 0.577; S4 Fig) when considering all fire-related treatments together. There was no significant *fire cue x fire frequency* interaction affecting the percentage of seed germination (*χ*^*2*^ = -20.68, *P* = 0.812; Fig 4A) and survival (*χ*^*2*^ = -2.24, *P* = 0.856; S4 Fig) of *H*. *aromaticum* populations. Fire frequency was positively correlated with seed pubescence (Pearson *r* = 0.68, P = 0.029) and dormancy (Spearman *R* = 0.64, *P* = 0.043), and negatively correlated with length-to-width ratio (*r* = -0.79, *P* = 0.006) of the seeds of *H*. *aromaticum* populations collected in 2013.

The phenotypic selection analysis performed on seeds from Lampa population showed that dormancy had a negative linear relationship to the probability of germination after 80°C and 100°C (β < 0; Table 2). Length-to-width ratio was positively related to seed survival at 100°C (Table 2); hence more elongated seeds had more chance to survive than rounded ones. When quadratic gradients were analyzed, dormancy showed disruptive selection at 100°C with survival as the fitness measure (γii > 0; Table 2), indicating that both higher and lower values of dormancy were favourable trait expressions. Pubescence and shape were subjected to disruptive selection under heat treatments when germination was the fitness measure (γii > 0; Table 2); that is, individuals with extreme values of these traits germinate more than individuals with intermediate values. On the other hand, seed pubescence and shape had negative correlational gradients on germination under heat-shock treatments (γij < 0; Table 2). Quadratic and correlational gradients together indicate that the probability of germination after heat shock is higher for elongated glabrouscent seeds or rounded pubescent seeds in relation to other trait combinations.

**Table 2:** Phenotypic selection analyses on Lampa population of *H*. *aromaticum*.

	Linear gradient		Quadratic gradient		Correlational gradient			
					Character j			
					Pubescence		Shape	
Character i	β_I_ (SE)	P	γ_ii_ (SE)	P	γ_ij_ (SE)	P	γ_ij_ (SE)	P
**Germination at 100°C**								
**Pubescence**	-0.21 (0.19)	0.292	**0.42 (0.13)**	**0.003**				
**Shape**	0.40 (0.20)	0.068	0.22 (0.12)	0.061	**-0.43 (0.18)**	**0.022**		
**Dormancy**	**-1.10 (0.21)**	**<0.001**	-0.30 (0.24)	0.223	0.35 (0.20)	0.089	-0.15 (0.25)	0.523
**Survival at 100°C**								
**Pubescence**	0.05 (0.22)	0.795	0.04 (0.19)	0.836				
**Shape**	**0.76 (0.24)**	**0.002**	-0.04 (0.17)	0.794	-0.17 (0.28)	0.560		
**Dormancy**	-0.11 (0.20)	0.586	**0.62 (0.30)**	**0.048**	-0.24 (0.24)	0.318	0.03 (0.31)	0.924
**Germination at 80°C**								
**Pubescence**	0.01 (0.17)	0.974	**0.28 (0.12)**	**0.027**				
**Shape**	-0.13 (0.17)	0.459	**0.27 (0.11)**	**0.016**	**-0.42 (0.20)**	**0.037**		
**Dormancy**	**-1.26 (0.17)**	**<0.001**	-0.10 (0.20)	0.618	-0.03 (0.18)	0.882	-0.30 (0.22)	0.184
**Survival at 80°C**								
**Pubescence**	-0.18 (0.20)	0.366	-0.11 (0.13)	0.153				
**Shape**	-0.16 (0.20)	0.426	0.24 (0.19)	0.206	-0.14 (0.19)	0.448		
**Dormancy**	0.21 (0.18)	0.234	0.24 (0.25)	0.353	-0.28 (0.17)	0.116	-0.35 (0.19)	0.071

Significant regression slopes are highlighted in bold (P<0.05, Generalized Linear Model, n = 68 individuals).

## Discussion

Seed pubescence and shape in *H*. *aromaticum* modulated seed responses to fire cues (heat shock and smoke) across populations and across individuals within a population, and these traits were correlated with fire frequency in natural habitats. These results confirm the idea proposed by Gómez-González *et al*. [28] of rapid evolution of seed traits driven by anthropogenic fires, and deepen into the mechanisms implicated in this process by showing that heat shock and smoke are involved in the adaptive responses. Specifically, we found that populations with rounded or pubescent seeds were, in general, more resistant to the effects of heat shock (80°C) and smoke than populations with elongated or glabrouscent seeds. Consistently, populations subjected to high fire frequency had, on average, more rounded and pubescent seeds than those from less frequently burned habitats. Similar fire frequency-trait correlations were already showed by Gómez-González *et al*. [28] using the same traits and populations in 2009, four years before our traits measures (in 2013).This also sustains the idea that seed pubescence and shape are heritable traits with the potential to evolve with fire (heritability was previously demonstrated by the parent-offspring regression method in a single population; [28]), since the pattern has been maintained throughout four generations and across populations. Interestingly, Gómez-González *et al*. [28] also showed that these traits were not correlated with an estimate of site productivity, and they suggested that fire might be more relevant in shaping pubescence and shape than other environmental factors such as nutrient or water availability. Our results bear out this hypothesis of fire-driven evolution, since in our phenotypic selection experiment, these traits were associated with fitness measures (germination and survival) when seeds were subjected to the specific signals of fire (heat shock and smoke).

It shall be stressed that, although recent fires seem to be changing the expression of seed traits in *H*. *aromaticum* populations, we cannot consider this species as fire-adapted because fire cues had overall negative effects on both seed germination and survival of the populations. In fire-adapted annuals from other MTC regions, dormancy is broken by heat and/or smoke and some species even depend on fire for germination to occur [10, 20]. By contrast, heat and smoke treatments did not break seed dormancy in *H*. *aromaticum* populations (independently on the fire frequency of their habitats), and within Lampa population specifically, individuals with higher level of dormancy did not show more germination after heat shock treatments. But, on the other hand, fire frequency was positively related with dormancy across populations. This suggests that other environmental factors that are tied to fire might modulate seed dormancy in this species (i.e., light, water, allelopathy [48]).

Our experimental approach is a strong simplification on how fire affects the seeds in nature, where multiple factors interact with each other. For example, fire frequency reduces fire severity by decreasing fuel load [49], which could have obscured our results on the relationship between fire frequency and germination responses to heat shock. Furthermore, vegetation patchiness in the Chilean matorral generates a spatial variability of fuel (dead grasses in open areas and dense tree litter beneath canopies), affecting the distribution of soil temperatures during fire [44]. Notwithstanding this, the fact that the matorral is mostly subjected to surface fires of moderate severity (even in closed mature communities) and that *H*. *aromaticum* generally colonizes open patches and microhabitats (S. Gómez-González, pers. obs.) probably have reduced the frequency-severity interaction in our study. On the other hand, the relationship of fire frequency with seed pubescence and shape across populations is consistent with the relationship of these traits with fitness measures in our phenotypic selection experiment, suggesting that fire frequency is an accurate estimate of the selective pressure imposed by fire in this system.

Correlational selection is an evolutionary mechanism that favours the expression of specific combinations of traits that are functional against the same selective pressure [30]. This type of natural selection can promote genetic correlation between characters and phenotypic integration as an adaptive consequence [50]. Our results of quadratic and correlational gradients together indicated that either elongated glabrouscent seeds or rounded pubescent seeds have the highest probability of germinating after heat-shock treatments (80°C and 100°C). To our knowledge, this is the first evidence suggesting correlational selection by fire on seed traits, where only certain trait combinations were adaptive against high temperatures. However, we found at the landscape scale that elongated glabrouscent seeds are less common in populations subjected to frequent fires. A feasible explanation for this pattern is that other selective forces might be acting against the expression of this specific trait combination just after fire (before germination), thus increasing only the frequency of rounded and pubescent phenotypes. For example, glabrouscent (elongated or rounded) seeds might be more susceptible to be attacked by micro-invertebrates or to be desiccated after fire than hairy ones, since the ecological function of seed pubescence has been mainly related to defence [51, 52] and the maintenance of water balance [53, 54].

The ecological explanation on why the combination of an elongated shape and low pubescence in the seed phenotype increased germination after heat shock is difficult to achieve. Similarly, Gómez-González *et al*. [28] found more seedling emergence of elongated seeds, in relation to rounded ones, after experimental litter burning in the same population. By contrast, Ruprecht *et al*. [33] showed that rounded seeds were more tolerant to experimental burning than elongated or flat seeds when analysing 37 species from temperate grasslands, and they appealed to the smaller relative surface (surface-to-volume ratio) that rounded seeds would have, increasing their heat tolerance. In *H*. *aromaticum*, selection on unmeasured traits that may be correlated with seed shape could explain why elongated glabrouscent seeds are favoured after heat shock [45].

To our knowledge, no studies have linked seed pubescence with the resistance to heat shock as we are showing here. But interestingly, Barthlott [55] suggested that the hairs on the surface of seeds could act as a mechanism of thermal insulation, as it occurs in the leaves and other parts of the plant body [56]. Since *H*. *aromaticum* seeds (achenes) are released in summer, they are exposed to high irradiance and drought. In addition, pubescent seeds can easily float with the surface runoff after the first rains in autumn (S. Gómez-González, pers. obs.), and thus disperse secondarily away from the mother plant. It might then be concluded that seed pubescence in *H*. *aromaticum* is an adaptation to high irradiance, water loss and/or water dispersal, and that its association with fire has therefore an exaptive nature. Of course, this trait is probably the result of a combination of selective pressures that converge in its habitat (summer drought, high irradiance, seed predation and, more recently, recurrent fire). However, independently of the original force that formed the first hairs on the achene surface of *H*. *aromaticum*, our results together with those of Gómez-González *et al*. [28] strongly suggest that anthropogenic fires have been re-shaping this trait in the last centuries, so we can consider this as the beginning of a fire adaptation (*sensu* Keeley *et al*. [7]). Although fire adaptations in the Chilean matorral are rare compared to other MTC regions [10], recent anthropogenic fires seem to be capable of driving adaptive evolution, particularly in short-lived, annual plants [28, 57]. However, it remains to be seen how seed shape and pubescence operate together to protect seeds from heat and smoke, and how general this pattern is in other ecosystems.

More research needs to be done in order to address whether fire is generating adaptations in seed traits of other plant species. This would be of high ecological relevance because fire-selected seed traits might also favour the evolution of other plant traits if fire remains prevalent compared to other selection forces. For instance, fire stimulated seed germination is central in the selection of seeder and resprouter life-forms [58] and it also seems to favour the evolution of flammability enhancing traits in woody plants from fire-prone habitats [16, 59]. The emerging studies during the last years on the evolutionary ecology of fire are demonstrating that this is a strong selection force that is rapidly changing plant traits at an ecological time scale [13, 16, 17, 28]. In ecosystems where fire has been recently introduced or enhanced by humans, the evolutionary effects on plants can be noted in short periods of time. For example, in the Canary Islands, Climent *et al*. [60] found positive correlation between bark thickness and serotiny in *Pinus canariensis* with the fire frequency registered in a period of 30 years. Anthropogenic fires caused by Native Americans in California have been related with recent changes in bark thickness in *P*. *radiata* and *P*. *muricata* [61]. In the same way, Gómez-González *et al*. [28] and this study strongly suggest the idea of rapid evolution of seed traits by anthropogenic fires in the last decades.

## Concluding remarks

Seed pubescence and shape are heritable traits that modulate adaptive responses to fire cues, which highlight the role of fire as a strong evolutionary agent. The frequency of human-made fires is increasing across ecosystems and biomes [62], and thus, the study of the role of fire as a selective force should no longer be restrained to natural fire-prone ecosystems. Anthropogenic fire is now a global scale disturbance that is affecting plant regeneration and vegetation structure in different types of habitats [63, 64], and the evolutionary consequences at the global scale has not yet been dimensioned. Therefore, anthropogenic processes affecting fire regimes should be included as a relevant part of the ecological theory of fire.

## Supporting Information

S1 Fig Interactive effects of fire cues and seed pubescence on the percentage of survival of *H*. *aromaticum* populations. Crosses represent plant individuals and there is one regression line per population (with different colours). Population codes in the box are in decreasing order of fire frequency (see codes in Fig 1 legend).
(TIF)

S2 Fig Interactive effects of fire cues and seed shape (length-to-width ratio) on the percentage of survival of *H*. *aromaticum* populations. Crosses represent plant individuals and there is one regression line per population (with different colours). Population codes in the box are in decreasing order of fire frequency (see codes in Fig 1 legend).
(TIF)

S3 Fig Relationship between the level of dormancy of *H*. *aromaticum* populations and the percentage of germination after fire cues (80°C, 100°C and smoke). Crosses represent plant individuals and there is one regression line per population (with different colours). Population codes in the box are in decreasing order of fire frequency (see codes in Fig 1 legend).
(TIF)

S4 Fig Interactive effects of fire cues and fire frequency on the percentage of survival of *H*. *aromaticum* populations. Dots represent the mean value of populations and error lines are 2SE. There is one regression line per treatment (different colours).
(TIF)

S1 File Excel file with the raw data. (XLS)

S1 Table Description of the 10 study sites, located in the Metropolitan County (Central Chile) and where the seeds of *H*. *aromaticum* were collected. (a) Number of fires per year, in 26 years (extracted from Gómez-González *et al*. 2011). (b) Personal observation of land owners and managers in relation to the fire history of the sites (modified from Gómez-González *et al*. 2011).
(DOC)

S2 Table Correlation matrix for the seed traits included in the phenotypic selection analysis. Significant correlations between traits are highlighted in bold (P < 0.05). There is no colinearity between pairs of traits (r< 0.8 in all cases, Pearson correlations, n = 67 individuals).
(DOC)
