# Supplementary figures and images for: Seed Pubescence and Shape Modulate Adaptive Responses to Fire Cues
Source: PLoS One. 2016 Jul 20;11(7):e0159655. doi: 10.1371/journal.pone.0159655 (PMC4954725; doi:10.1371/journal.pone.0159655)

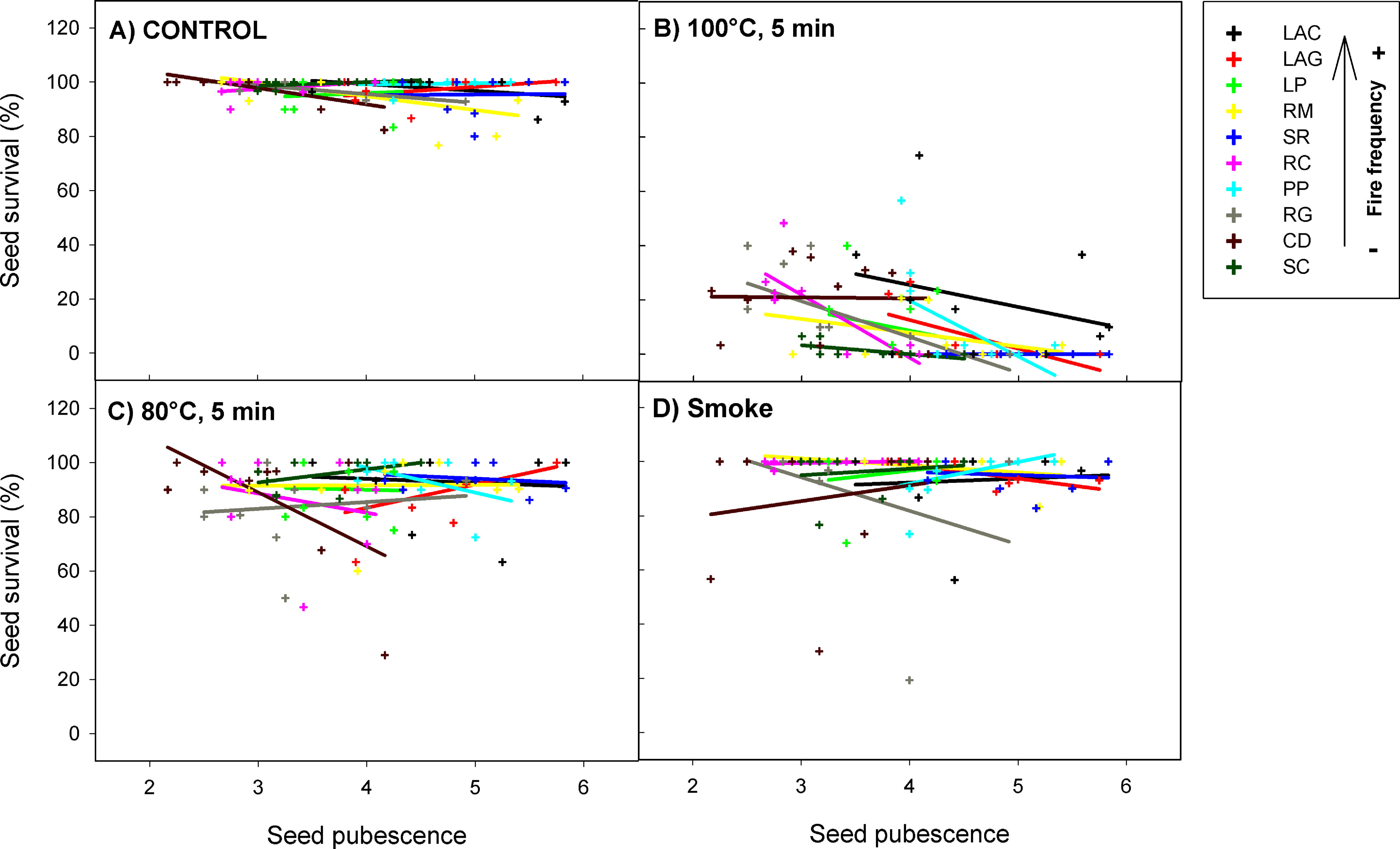

Supplement: S1 Fig — Crosses represent plant individuals and there is one regression line per population (with different colours). Population codes in the box are in decreasing order of fire frequency (see codes in Fig 1 legend). (TIF) [file pone.0159655.s001.tif]

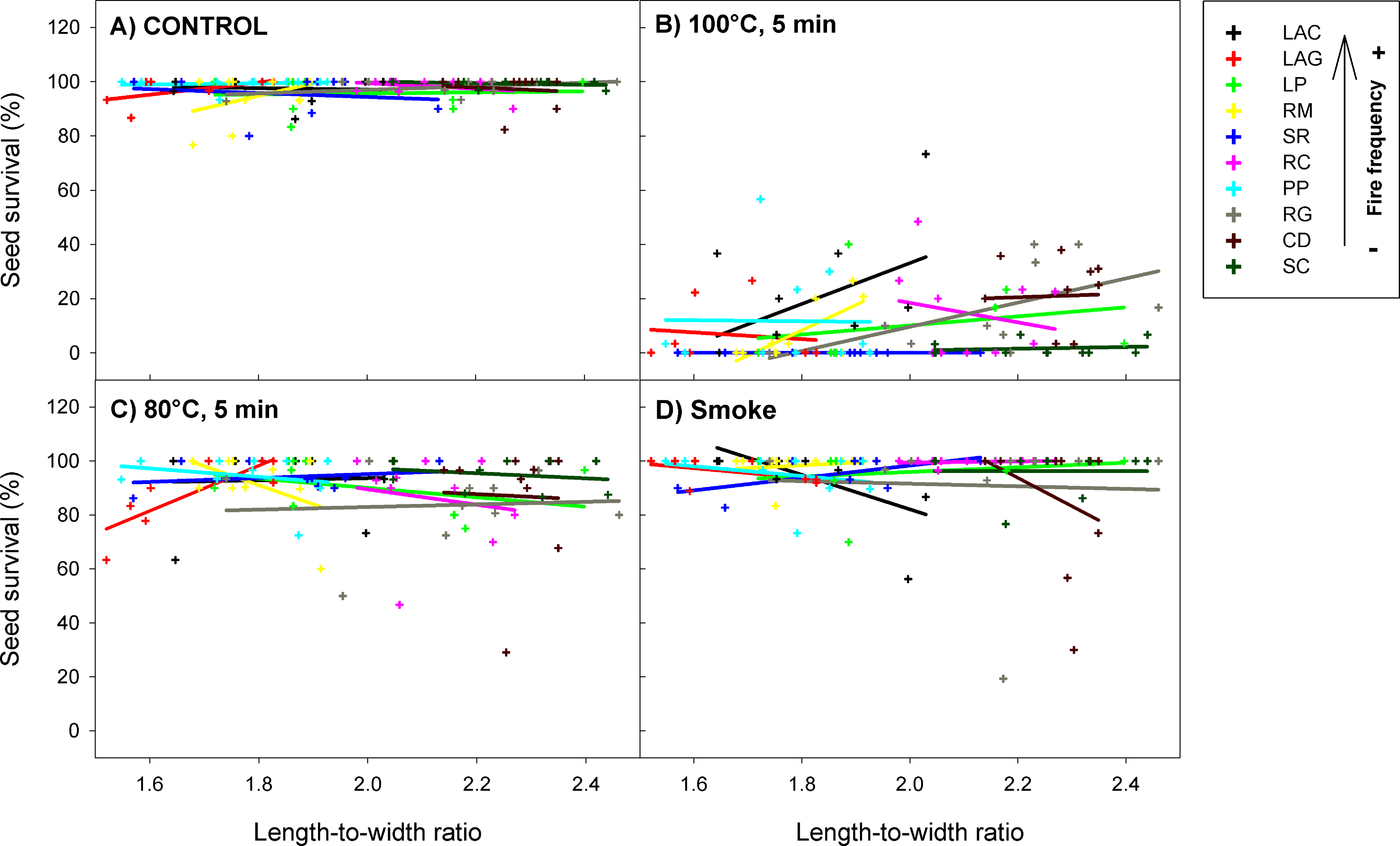

Supplement: S2 Fig — Crosses represent plant individuals and there is one regression line per population (with different colours). Population codes in the box are in decreasing order of fire frequency (see codes in Fig 1 legend). (TIF) [file pone.0159655.s002.tif]

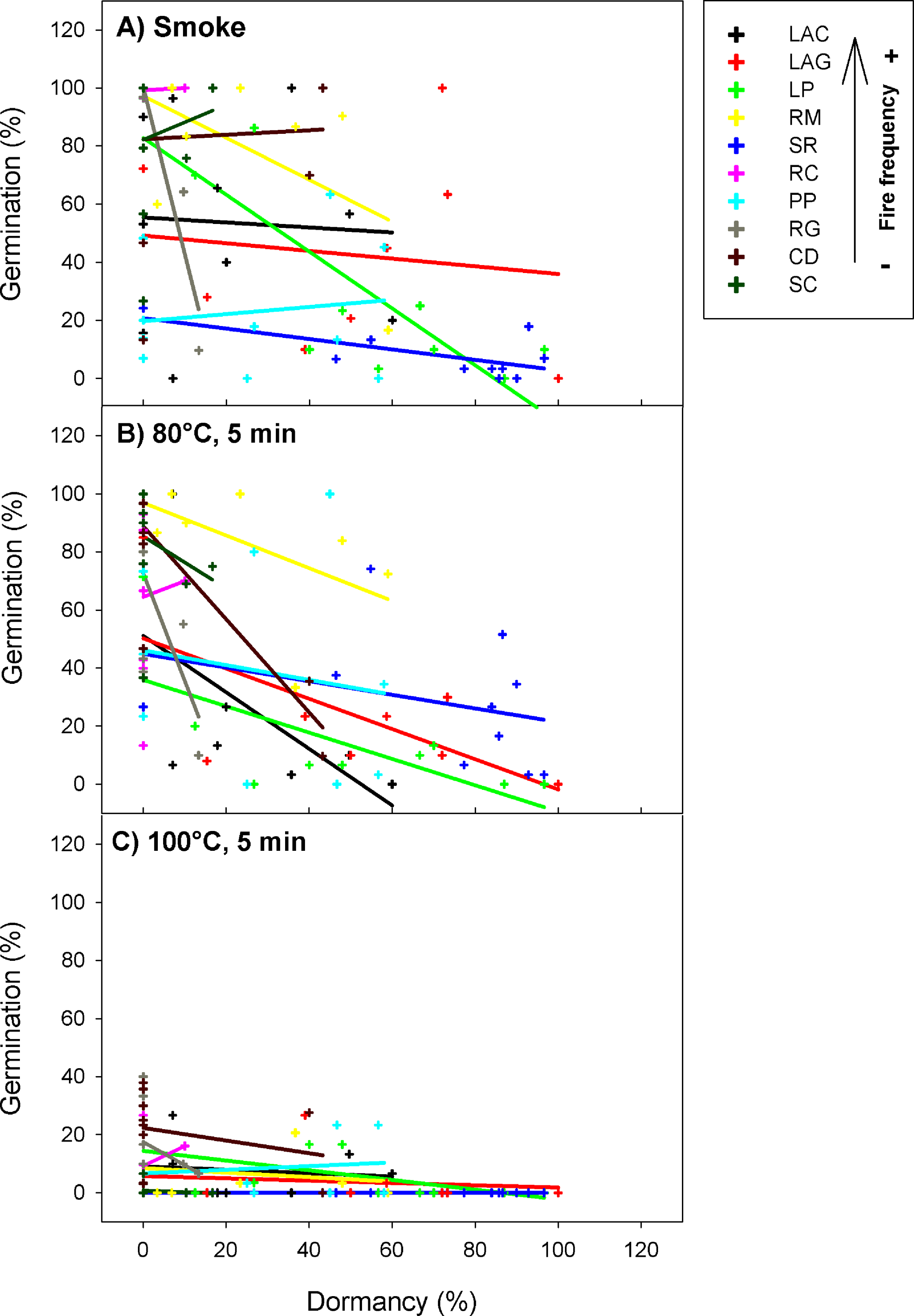

Supplement: S3 Fig — Crosses represent plant individuals and there is one regression line per population (with different colours). Population codes in the box are in decreasing order of fire frequency (see codes in Fig 1 legend). (TIF) [file pone.0159655.s003.tif]

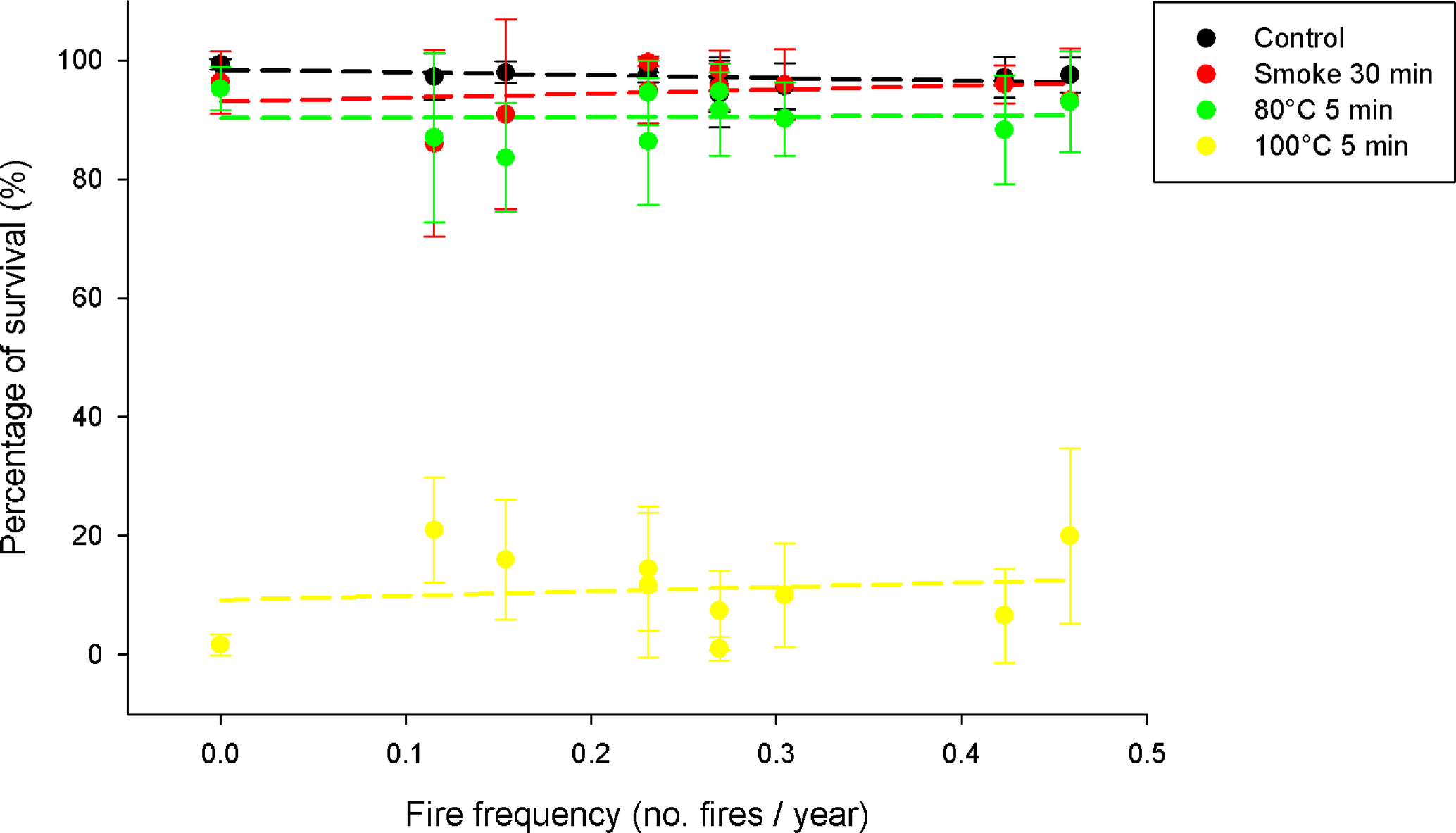

Supplement: S4 Fig — Dots represent the mean value of populations and error lines are 2SE. There is one regression line per treatment (different colours). (TIF) [file pone.0159655.s004.tif]
