# Supplementary material for: Seed Pubescence and Shape Modulate Adaptive Responses to Fire Cues
Source: PLoS One. 2016 Jul 20;11(7):e0159655. doi: 10.1371/journal.pone.0159655 (PMC4954725; doi:10.1371/journal.pone.0159655)
Supplement: S1 Table — (a) Number of fires per year, in 26 years (extracted from Gómez-González et al. 2011). (b) Personal observation of land owners and managers in relation to the fire history of the sites (modified from Gómez-González et al. 2011). (DOC) [file pone.0159655.s006.doc]

S1 Table:

| Code | Locality name | Political district | Coordinates | Altitude | Fire frequency ^(a)^ | Fire return interval (years) | Additional information ^(b)^ |
| --- | --- | --- | --- | --- | --- | --- | --- |
| SC | San Carlos de Apoquindo | Las Condes | S33°24´9´´ W70°29´1´´ | 1,140 | 0 | Unburned | Protected Natural Park. No fires in the last 70–80 y. |
| CD | Cuesta La Dormida | Til Til | S33°4´26´´ W70°56´38´´ | 660 | 0.11 | 9.1 | Well conserved private land. Only one fire in the last 40–50 y. |
| RG | Rungue | Til Til | S33°0´26´´ W70°53´52´´ | 713 | 0.15 | 6.7 | Private land used for grazing. One fire recorded in 2007. |
| RC | Río Clarillo | Pirque | S33°43´37´´ W70°29´11´´ | 911 | 0.23 | 4.3 | Natural Reserve protected since the 1980s. One fire 11 y ago. Frequently burned before 80´s to produce charcoal. |
| PP | Punta Peuco | Til Til | S33°5´53´´ W70°49´50´´ | 569 | 0.23 | 4.3 | Private land used for grazing and rounded by crops. Several fires in the 1990s. |
| RM | Rinconada Maipú | Maipú | S33°30´5´´ W70°52´48´´ | 481 | 0.27 | 3.7 | Private land used for grazing. At least one fire in the last 10 y. |
| SR | Quebrada San Ramón | La Reina | S33°26´2´´ W70°30´21´´ | 895 | 0.27 | 3.7 | Protected Park. One or two fires in the last 20 y. Frequently burned in the past to produce charcoal. |
| LP | Lampa | Lampa | S33°15´34´´ W70°54´3´´ | 560 | 0.30 | 3.3 | Disturbed area (grazing, bonfires, etc). Open access near the road. Frequently burned. |
| LAG | Los Aromos (grazing area) | Til Til | S33°6´26´´ W70°52´41´´ | 570 | 0.42 | 2.4 | At least two fires in the last 10 y. There were probably frequent fires in the past. |
| LAC | Los Aromos (crop area) | Til Til | S33°5´17´´ W70°51´13´´ | 560 | 0.46 | 2.2 | Area burned every two-three years. Patches of vegetation very sparse. |
