# Supplementary material for: Seed Pubescence and Shape Modulate Adaptive Responses to Fire Cues
Source: PLoS One. 2016 Jul 20;11(7):e0159655. doi: 10.1371/journal.pone.0159655 (PMC4954725; doi:10.1371/journal.pone.0159655)
Supplement: S2 Table — Significant correlations between traits are highlighted in bold (P < 0.05). There is no colinearity between pairs of traits (r< 0.8 in all cases, Pearson correlations, n = 67 individuals). (DOC) [file pone.0159655.s007.doc]

S2 Table

|  | **Pubescence** | | **Shape (lengh:width)** | | **Dormancy (%)** | |
| --- | --- | --- | --- | --- | --- | --- |
|  | r | P | r | P | r | P |
| **Pubescece** | - | - | **-0.46** | **<0.001** | 0.17 | 0.173 |
| **Shape (length:width)** |  |  | - | - | -0.15 | 0.209 |
| **Dormancy (%)** |  |  |  |  | - | - |
